# Physically Inactive Undergraduate Students Exhibit More Symptoms of Anxiety, Depression, and Poor Quality of Life than Physically Active Students

**DOI:** 10.3390/ijerph20054494

**Published:** 2023-03-03

**Authors:** Endrew Eduardo Santos de Santana, Lucas Melo Neves, Karla Cardoso de Souza, Tassia Barcelos Mendes, Fabricio Eduardo Rossi, Ariana Aline da Silva, Rosemeire de Oliveira, Mauro Sergio Perilhão, Hamilton Roschel, Saulo Gil

**Affiliations:** 1 Programa de Atividades Esportivas Extensivas à Comunidade—PAEC, Santo Amaro University, São Paulo 04829-300, Brazil; 2 Post-Graduate Program in Health Sciences, Santo Amaro University, Sao Paulo 04829-300, Brazil; 3 Bipolar Disorder Program (PROMAN), Department of Psychiatry, School of Medicine FMUSP, University of São Paulo, São Paulo 05403-000, Brazil; 4 Immunometabolism of Skeletal Muscle and Exercise Research Group, Department of Physical Education, Federal University of Piauí, Teresina 64049-550, Brazil; 5 Applied Physiology and Nutrition Research Group, School of Physical Education and Sport, Rheumatology Division, School of Medicine FMUSP, Universidade de Sao Paulo, São Paulo 14040-901, Brazil

**Keywords:** physical activity, mental disorders, students

## Abstract

Background: The World Health Organization (WHO) recommends at least 150 min of moderate or vigorous activity (MVPA) per week for health benefits. However, meeting WHO guidelines for physical activity has been shown to be a great challenge for general populations and it may be even more difficult for undergraduate students due to elevated academic demand, thus negatively affecting general health status. Thus, this study investigated whether undergraduate students meeting WHO guidelines for physical activity show greater scores for symptoms of anxiety, depression, and poor quality of life than their counterparts not meeting guideline recommendations. Additionally, symptoms of anxiety, depression, and poor quality of life among academic areas were compared. Methods: This is a cross-sectional study. The participants were recruited through messaging apps or institutional e-mail. The participants filled out an online consent form, questionnaires to assess demographic and academic characteristics, the International Physical Activity Questionnaire, the Beck depression and anxiety inventory, and the short-form 36-item health survey questionnaire. The participants were classified as physically active (MVPA > 150 min/week) or inactive (MVPA < 150 min/week) according to WHO Guidelines. Results: A total of 371 individuals were included in the analysis. Physically inactive students demonstrated higher scores of depression (17.96 vs. 14.62; 95% CI: −5.81 to −0.86; *p* = 0.0083) than physically active ones. SF-36 analyses revealed that physically inactive students had lower values in mental (45.68 vs. 52.77; 95% CI: 2.10 to 12.06; *p* = 0.0054) and physical (59.37 vs. 67.14; 95% CI: 3.24 to 12.30; *p* = 0.0015) domains compared with physically active ones. As for SF-36 subscales, physically inactive students showed lower scores in function capacity (70.45 vs. 79.70; 95% CI: 4.27 to 14.49; *p* = 0.0003), mental health (45.57 vs. 55.60; 95% CI: 5.28 to 14.76; *p* < 0.0001), social aspects (48.91 vs. 57.69; 95%CI: 3.47 to 14.08; *p* = 0.0012), vitality (42.19 vs. 50.61; 95% CI: 3.47 to 13.35; *p* = 0.0009), pain (61.85 vs. 68.00; 95% CI: 1.27 to 11.02; *p* = 0.0135), and general health status (53.82 vs. 63.81; 95% CI: 5.21 to 14.75; *p* < 0.0001) than their physically active peers. Conclusions: The findings suggest that undergraduate students who do not meet WHO guidelines for physical activity display higher scores of anxiety, depression, and poor quality of life in comparison with their counterparts meeting physical activity guidelines. Collectively, these data suggest the need for academic institutions and policy makers to monitor and promote in-campus interventions to encourage physical activity.

## 1. Introduction

Symptoms of anxiety, depression, and, consequently, poor quality of life are commonly observed in distinct settings such as social, work, and academic settings [1,2,3]. In particular, undergraduate students are part of a specific social group that often experience elevated amounts of stress imposed by the academic environment, studying, examinations, and teacher or parental pressure, which may negatively impact lifestyle, emotional, and health outcomes [3,4].

World Health Organization (WHO) guidelines on physical activity recommend 150 min of moderate-to-vigorous activity (MVPA) per week to provide health benefits [5]. Previous studies have demonstrated that individuals that meet WHO guidelines for physical activity show lower symptoms of depression and anxiety [6,7] when compared to their counterparts that do not meet the recommendations. However, meeting WHO recommendations for physical activity has not been shown to be an easy task for the general population [8], which may be even more critical for undergraduate students due to the elevated academic demand. Indeed, there is evidence showing an elevated prevalence of physical inactivity among undergraduate students [9,10], but it is not a consensus [11]. De Souza et al. [9] observed a prevalence of physical inactivity of 67% and 34% in medicine and physical education students, respectively. Similarly, other studies have also reported insufficient physical activity levels in undergraduate students and they have been shown to be associated with health outcomes such as depression and daytime sleepiness [12,13,14]. In this scenario, it is reasonable to speculate that physically inactive undergraduate students may show mental disorders and, consequently, poor quality of life. However, this assumption is not completely elucidated. Moreover, Ireña et al. [15] suggested that students of the faculty of sports have better physical fitness compared to physiotherapy students, suggesting that students who have physical activities within the curriculum or disciplines discussing the risks or benefits of physical activity (e.g., medicine and physical education) may show different physical activity levels and, consequently, distinct health-related outcomes in comparison with areas which do not study aspects related to physical activity (e.g., computer sciences and engineering).

Therefore, the aim of this study was to investigate whether undergraduate students meeting WHO guidelines for physical activity show greater scores for symptoms of anxiety, depression, and poor quality of life in comparison with their counterparts not meeting the recommendations. Additionally, physical activity level and symptoms of anxiety, depression, and poor quality of life were compared among different academic areas.

## 2. Materials and Methods

### 2.1. Study Design and Participants

This is a cross-sectional study conducted in the University Santo Amaro (Sao Paulo, Brazil) between May 2022 and July 2022. Participants were recruited through messaging apps or institutional e-mail. All participants filled out an online consent form (Google forms^®^), questionnaires on demographic and academic characteristics, the International Physical Activity Questionnaire, the Beck depression and anxiety inventory, and the short-form 36-item health survey questionnaire.

Men and women aged ≥18 years currently enrolled at university were eligible to participate in the study. The exclusion criteria were as follows: medical conditions precluding physical activities and/or affecting the ability to complete questionnaires. This study was approved by the local ethics committee (CAAE: 32333420.4.0000.0081).

### 2.2. International Physical Activity Questionnaire

Physical activity was assessed using the International Physical Activity Questionnaire-Short Form (IPAQ) [16]. In brief, IPAQ inquires about physical activity (i.e., walking, moderate-intensity activity, and vigorous-intensity activity) in the past 7 days. The time spent in moderate and vigorous activity (MVPA) was calculated as the number of days multiplied by the number of hours reported. The participants were classified as physically active (MVPA > 150 min/week) or inactive (MVPA < 150 min/week) according to WHO guidelines.

### 2.3. Anxiety and Depression Symptoms Assessment

The anxiety and depression symptoms were assessed using the Beck anxiety inventory [17] and Beck depressive inventory [18], which are composed of 21 multiple-choice statements, each with 4 possible answers (0–3). The final score ranges from 0 to 63 points. Participants who scored >25 for the Beck anxiety inventory and >35 for Beck depression inventory were classified as severe.

### 2.4. Health-Related Quality of Life

Health-related quality of life was assessed using the short-form 36-item health survey questionnaire (SF-36) [19,20]. The SF-36 questionnaire is a multipurpose, self-administered short-form health survey containing 36 items divided into 8 domains: functional capacity, physical aspects, pain, general health, vitality, social aspects, emotional aspects, and mental health. The physical domain refers to a sum of scores of functional capacity, physical aspects, pain, and general health divided into 4; the mental domain consists of a sum of vitality, social aspects, emotional aspects, and mental health divided into 4. It provides a general score from 0 to 100, with lower scores indicating worse condition [21]. Participants who scored <50 in mental and physical domain were labelled as poor quality of life.

### 2.5. Statistical Analyses

Sample size was determined with the aid of an R package *pwr* (version 1.3-0). The analysis was conducted by inputting α error (0.05), power (1–β error = 0.95), and assuming a moderate effect size (Cohen’s *d*) of 0.4. Calculations were based on an independent t-test and the total sample size returned was 326 individuals.

Data are presented as absolute (n) and relative (%) frequencies, means ± SD. Independent t-tests were performed to test possible between-group differences (physically active vs. physically inactive) for all dependent variables. One-way ANOVA was performed to compare MVPA and scores of anxiety, depression, and quality of life among academic areas (humanities vs. natural and applied sciences vs. social sciences). Whenever a significant F-value was detected, the Tukey post hoc test was used for multiple comparisons. Possible between-group differences in the frequency of severe symptoms of depression, anxiety, and poor quality of life in the mental and physical domain were tested using a Fisher exact test. The significance level was set to *p* ≤ 0.05. All analyses were performed in the statistical environment R (version 3.5.3; R Core Team 2020).

## 3. Results

**Figure 1:**
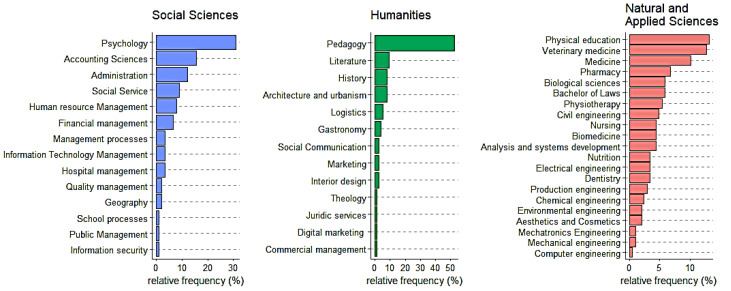
Relative frequency of respondents from each course.

A total of 371 individuals responded to the call and were included in the analysis. The relative frequency of respondents of each course and the characteristics of these participants may be checked in detail in Figure 1 and Table 1, respectively. Overall, the sample comprised individuals of both sexes (77% female) aged 31 ± 9 years. Ten percent of the participants were current smokers. The prevalence of obesity, depression, hypertension, type 2 diabetes, dyslipidemia, and asthma were 19%, 13%, 6%, 2%, 2%, and 2%, respectively. Only 35% of the participants met the physical activity recommendations.

**Figure 2:**
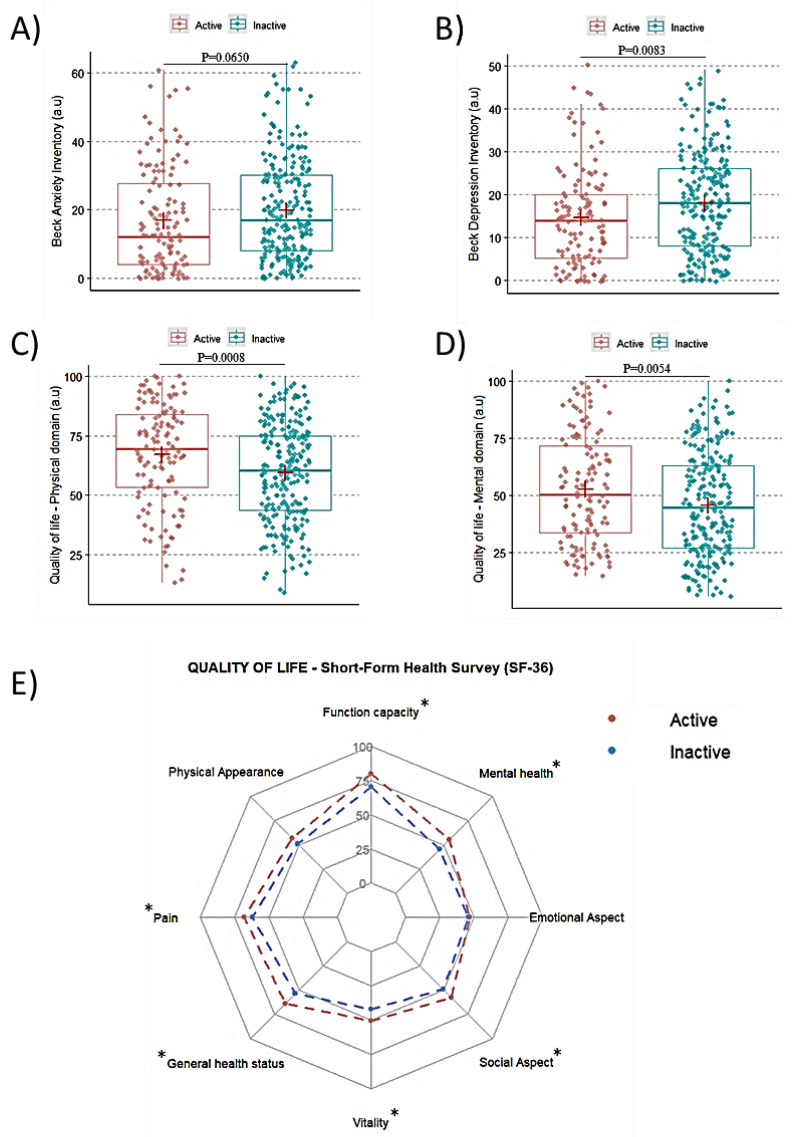
Scores of anxiety, depression, and quality of life in physically active and inactive students. Panel (**A**): Scores obtained in Beck anxiety inventory; Panel (**B**): Scores obtained in Beck depression inventory; Panel (**C**): Scores of quality of life in the physical domain; Panel (**D**): Scores of quality of life in mental domain; Panel (**E**): Radar plot of quality of life in each domain; * Indicates *p* < 0.05.

Physically inactive undergraduate students demonstrated higher scores of depression (17.96 vs. 14.62; 95% CI: −5.81 to −0.86; *p* = 0.0083) than physically active ones (Figure 2A,B). Additionally, physically inactive students showed higher scores of anxiety in comparison with physically active ones, but this difference did not reach statistical significance (19.83 vs. 16.80; 95% CI: −6.24 to 0.19; *p* = 0.0650).

The SF-36 analyses revealed that physically inactive students had lower values in the mental (45.68 vs. 52.77; 95% CI: 2.10 to 12.06; *p* = 0.0054) and physical (59.37 vs. 67.14; 95% CI: 3.24 to 12.30; *p* = 0.0015) domains compared with physically active ones. As for the SF-36 subscales, physically inactive students showed lower scores in functional capacity (70.45 vs. 79.70; 95% CI: 4.27 to 14.49; *p* = 0.0003), mental health (45.57 vs. 55.60; 95% CI: 5.28 to 14.76; *p* < 0.0001), social aspects (48.91 vs. 57.69; 95% CI: 3.47 to 14.08; *p* = 0.0012), vitality (42.19 vs. 50.61; 95% CI: 3.47 to 13.35; *p* = 0.0009), pain (61.85 vs. 68.00; 95% CI: 1.27 to 11.02; *p* = 0.0135), and general health status (53.82 vs. 63.81; 95% CI: 5.21 to 14.75; *p* < 0.0001) in comparison with their active counterparts. No between-group differences were observed for emotional aspects or physical appearance (both *p* > 0.05) (Figure 2C–E).

**Figure 3:**
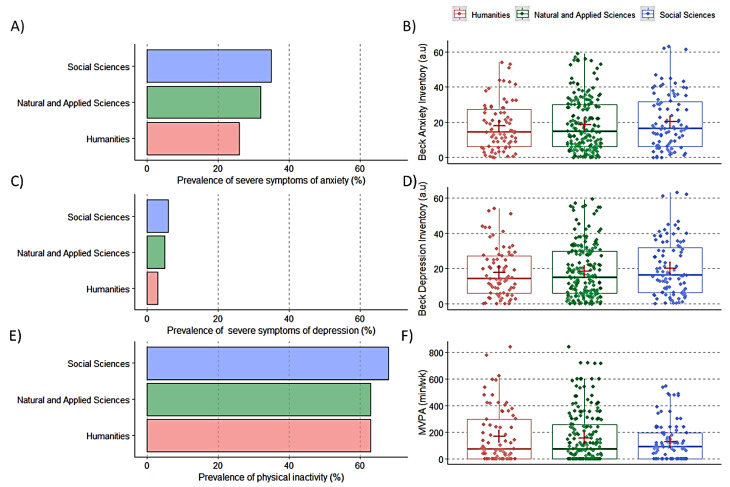
Scores of anxiety, depression, and physical inactivity according to academic areas. Panel (**A**): Prevalence of severe symptoms of anxiety (Score > 25); Panel (**B**): Scores obtained in Beck anxiety inventory; Panel (**C**): Prevalence of severe symptoms of depression; Panel (**D**): Scores obtained in Beck depression inventory (Score > 35); Panel (**E**): Prevalence of physical inactivity (<150 min/week of moderate-to-vigorous activity); Panel (**F**): Time spent in moderate to vigorous physical activity (MVPA).

The frequencies of students of social sciences, natural and applied sciences, and humanities reporting severe symptoms of anxiety were 35%, 32%, and 26%, respectively. For severe symptoms of depression, the prevalence was 6%, 5%, and 3%, respectively. The prevalence of physical inactivity for students of social sciences, natural and applied sciences, and humanities was 68%, 63%, and 63%, respectively. No between-academic-area differences were observed for the prevalence and scores of physical activity, anxiety, and depression (all *p* > 0.05)(Figure 3A–F).

**Figure 4:**
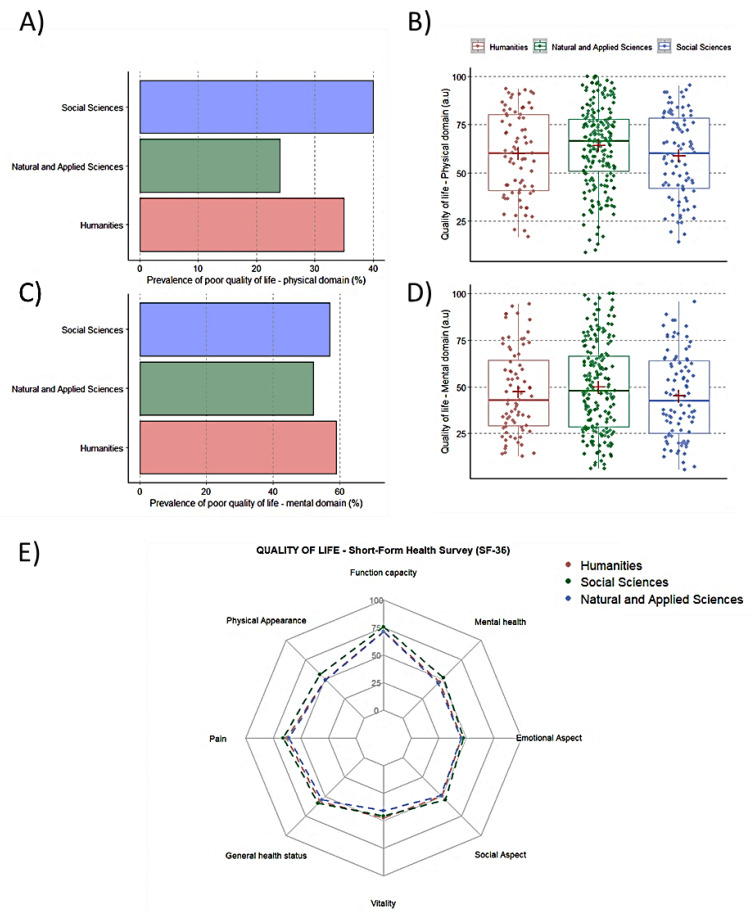
Scores of quality of life according to academic areas. Panel (**A**): Prevalence of poor quality of life in the physical domain (Score < 50); Panel (**B**): Scores of quality of life in the physical domain; Panel (**C**): Prevalence of poor quality of life in the mental domain (Score < 50); Panel (**D**). Scores of quality of life in the mental domain; Panel (**E**): Radar plot of quality of life in each domain.

The prevalence of students of social sciences, natural and applied sciences, and humanities reporting poor quality of life for the physical and mental domain was 40%, 24%, and 35%, and 57%, 52%, and 59%, respectively. No between-academic-area differences were verified for the prevalence of poor quality of life in both domains (both *p* > 0.05). The analysis of continuous data of SF-36 among academic areas revealed similar scores for all subscales and physical and mental domains (all *p* > 0.05)(Figure 4A–E).

## 4. Discussion

The main findings indicate that undergraduate students who do not meet WHO guidelines for physical activity exhibit higher scores of anxiety and depression and lower scores of quality of life in comparison with those meeting guideline recommendations. Additionally, an elevated prevalence of physical inactivity (>62%), severe symptoms of anxiety (>25%), and poor quality of life (physical domain: >23; mental domain: >51%) was observed, irrespective of academic area. This study suggests an inadequate lifestyle, with potential negative effects on health-related outcomes and quality of life, among undergraduate students of different academic areas.

Physical inactivity inarguably consists of a risk for distinct health-related outcomes such as mental [7,22,23] and cardiometabolic [24,25,26] disorders and, mostly important, morbimortality [27]. WHO guidelines recommend that adults should complete at least 150 min of MVPA per week to provide health benefits and mitigate risks related to physical inactivity [5]. Nonetheless, guideline recommendations have been proven hard to meet. Indeed, a pooled analysis of 358 population-based surveys with 1.9 million participants reported that 27% of the world’s population and 47% of Brazil’s population are, in fact, physically inactive [8]. Despite these alarming numbers, some specific groups (e.g., undergraduate students) may show an even higher prevalence of physical inactivity, which may still be more detrimental for health.

The results of the current study revealed a prevalence of 65% of physical inactivity in undergraduate students. Importantly, this elevated frequency of individuals with insufficient physical activity levels was observed regardless of academic area. These findings are in line with previous studies reporting elevated frequencies of students not meeting physical activity guidelines [28,29,30], which may negatively affect health-related parameters. Indeed, studies have demonstrated that physically inactive undergraduate students exhibit poor health-related outcomes such as sleep disorders [31,32], obesity [33], inadequate nutritional habits [34], and mental health issues [35]. These data extend the risk of physical inactivity to the health of undergraduate students since higher scores of anxiety, depression, and poor quality of life were also observed in physically inactive students than their physically active counterparts.

The association between physical activity, anxiety, and depression has been repeatedly reported [22,23,36]. Despite the fact that the mechanisms related to the negative impact of physical inactivity on mental and brain disturbances are not completely elucidated, some mediators have been suggested, such as impairments in neurogenesis, increased inflammatory profiles, and decreases in the hippocampal volume [37]. The hippocampus plays a pivotal role in the consolidation of information from short-term memory to long-term memory [38,39]. Thus, it is acceptable to speculate that physically inactive students reporting symptoms of anxiety and depression also display reduced hippocampal volume and, consequently, reduced academic performance. Moreover, it is noteworthy that anxiety and depression have been associated with dropout, poor academic self-concept [40], and lower academic achievement [41].

Medicine, physiotherapy, and kinesiology (i.e., natural and applied sciences) are some academic courses that include disciplines discussing the risk of an unhealthy lifestyle. Therefore, we expected that students with knowledge of the risks of not maintaining a healthy lifestyle might show elevated levels of physical activity levels and, consequently, good physical and mental health. However, the current findings do not support this assumption since we did not observe any statistically significant difference in physical activity levels, symptoms of anxiety, depression, or quality of life among distinct academic areas. This result is contrary to the suggestion from Ireña et al. [15] that students who partake in physical activities within the curriculum or disciplines discussing the risks or benefits of physical activity may show higher physical activity levels and, consequently, better health outcomes than students from areas that do not study aspects related to physical activity.

This study is not free of limitations. The observational cross-sectional design and the absence of adjusted analysis hampers establishing cause-and-effect relationships. The sample size was limited and, thus, some caution must be taken in the extrapolation of our results. All participants were enrolled in a single university and, thus, may not reflect the behavior of students at other higher education institutions. Physical activity levels were assessed through a questionnaire and reflected the week prior to follow-up assessments. Moreover, the use of questionnaires to assess physical activity is prone to recall bias and over-reporting.

## 5. Conclusions

In conclusion, the main findings of this study suggest that undergraduate students who do not meet WHO guidelines for physical activity display higher scores of anxiety, depression, and poor quality of life than physically active ones. Considering the potential impact of physical inactivity on health-related outcomes, we suggest the promotion of physical activity on campus in order to mitigate the potential negative effects of physical inactivity on health outcomes. In addition, these measures can reduce the elevated level of evasion from Brazilians in higher education institutes [42].

## Figures and Tables

**Table 1:** Characteristics of the participants.

Outcomes	n = 371
Age, years	31 ± 9
Sex, n (%)	
Female	287 (77%)
Male	84 (23%)
Ethnicity, n (%)	
White	56 (15%)
Black	189 (51%)
Asian	116 (31%)
Pardo ^a^	10 (3%)
Smoking status, n (%)	
Never	33 (9%)
Current	39 (10%)
Former smoker	299 (81%)
Comorbidities, n (%)	
Asthma	8 (2%)
Systemic arterial hypertension	22 (6%)
Depression	48 (13%)
Dyslipidemia	9 (2%)
Obesity (IMC ≥ 30 kg/m^2^)	72 (19%)
Type 2 diabetes	7 (2%)

^a^ = Pardo is the exact term used in Brazilian Portuguese, meaning “mixed ethnicity”, according to the Brazilian Institute of Geography and Statistics.

## Data Availability

All background information on individuals and information for participants included in this study are available from corresponding author on reasonable request.
